# Comparison of total and cytoplasmic mRNA reveals global regulation by nuclear retention and miRNAs

**DOI:** 10.1186/1471-2164-13-574

**Published:** 2012-10-30

**Authors:** Beata Werne Solnestam, Henrik Stranneheim, Jimmie Hällman, Max Käller, Emma Lundberg, Joakim Lundeberg, Pelin Akan

**Affiliations:** 1 KTH Royal Institute of Technology, Science for Life Laboratory (SciLifeLab Stockholm), School of Biotechnology, Division of Gene Technology, SE-171 65, Solna, Sweden; 2 KTH Royal Institute of Technology, Science for Life Laboratory (SciLifeLab Stockholm), School of Biotechnology, Division of Proteomics, SE-171 65, Solna, Sweden

**Keywords:** Differential detection, Gene expression, Nuclear retention, miRNA regulation, RNA-Seq

## Abstract

**Background:**

The majority of published gene-expression studies have used RNA isolated from whole cells, overlooking the potential impact of including nuclear transcriptome in the analyses. In this study, mRNA fractions from the cytoplasm and from whole cells (total RNA) were prepared from three human cell lines and sequenced using massive parallel sequencing.

**Results:**

For all three cell lines, of about 15000 detected genes approximately 400 to 1400 genes were detected in different amounts in the cytoplasmic and total RNA fractions. Transcripts detected at higher levels in the total RNA fraction had longer coding sequences and higher number of miRNA target sites. Transcripts detected at higher levels in the cytoplasmic fraction were shorter or contained shorter untranslated regions. Nuclear retention of transcripts and mRNA degradation via miRNA pathway might contribute to this differential detection of genes. The consequence of the differential detection was further investigated by comparison to proteomics data. Interestingly, the expression profiles of cytoplasmic and total RNA correlated equally well with protein abundance levels indicating regulation at a higher level.

**Conclusions:**

We conclude that expression levels derived from the total RNA fraction be regarded as an appropriate estimate of the amount of mRNAs present in a given cell population, independent of the coding sequence length or UTRs.

## Background

The advent of sequence-based assays of transcriptomes (RNA-Seq) has allowed better quantification of mRNA, with less bias and greater dynamic range than microarrays [1,2]. However RNA-Seq is undergoing a rapid evolution, and the impact of basic experimental design on data quality is still under investigation.

The majority of published transcriptome data have used RNA extracted from the whole cell (total RNA), assuming a negligible contribution of nuclear RNA to the total RNA population. This assumption has been challenged by Trask *et al.*[3], who demonstrated that the nuclear contribution does impact the gene expression profile when examining steady-state messenger RNA (mRNA) by microarray analysis. RNA extracted from the cytoplasmic fraction, which does not contain a nuclear RNA contribution, could also be used for RNA-Seq experiments. However, none of the available studies on RNA-Seq quantification have compared the use of total RNA with the use of cytoplasmic RNA.

During RNA synthesis, mRNAs are transcribed, spliced, capped, and polyadenylated in the nucleus and the resulting steady-state RNA is transported from nucleus to cytoplasm via nuclear pore complexes for translation. Messenger ribonucleoproteins are co-transcriptionally recruited to mRNA, and direct the export of mRNAs via their interaction with mRNA export factors and nuclear pore complexes [4]. This process is regulated at many levels, yielding a dynamic steady-state mRNA population that is maintained by synthesis and turnover, at varying rates for each individual transcript [5]. The rate of transportation from nucleus to cytoplasm can affect the amount of transcript detected in both the total and the cytoplasmic fractions, and hence might bias measurements of transcript levels. It has previously been shown that mRNA molecules that are not of immediate need to produce proteins are retained in the nucleus [6,7]. In addition to nuclear retention, the gene level is also regulated by other mechanisms and one of them is the degradation of mRNA by the exosome complex [8,9].

It is known that the levels of mRNA and protein abundance in cells are modestly correlated [10-12]. One can argue that cytoplasmic RNA is a better proxy for protein levels since the cytoplasmic fraction contains only mature RNA; unlike total RNA, which also contains nuclear RNA. Validation of this argument will require studies that assess how well the levels of total RNA and cytoplasmic RNA are correlated with protein abundance.

To investigate the impact of nuclear transcripts present in total RNA, we compared the expression levels of genes obtained from the total fraction with those obtained from the cytoplasmic fraction. We performed poly(A)+ RNA-Seq experiments on three human cancer cell lines (A-431, U-2 OS, and U-251MG) on cytoplasmic and total RNA fractions in quadruplicates. We investigated the effect of the length and structure of untranslated regions and the length of the coding sequences on the transcript levels in total and cytoplasmic RNA. miRNA-mediated degradation of transcripts and its role in transcript regulation was also investigated, as well as the effect on the correlation with protein levels. We present here an extensive study of RNA-Seq that compares gene expression levels from poly (A) isolated total and cytoplasmic RNA as well as their relation to protein levels.

## Results

**Figure 1:**
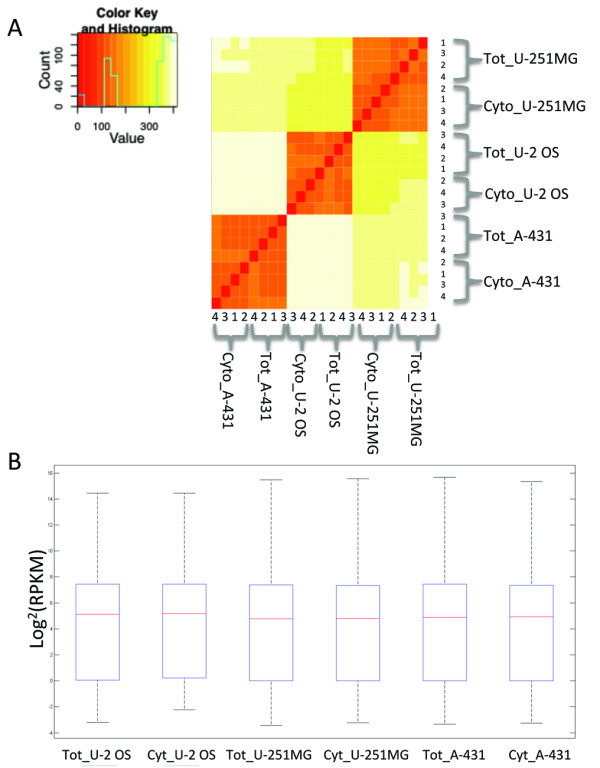
**Distribution of gene expressions for the total and cytoplasmic preparation. A**: Heatmap of sample preparation and cell lines. **B**: The distribution of all RPKM values for total and cytoplasmic RNA for the three cell lines U-2 OS, U-251MG, and A-431. The mean RPKM values for total and cytoplasmic RNA of each cell line did not differ significantly (*p* < 0.05).

RNA from three different human cell lines (A-431, U-2 OS, and U-251MG) was extracted from whole cells (total RNA) and from the cytoplasm (cytoplasmic RNA), which does not include nuclear transcripts. Each extraction was replicated four times. To ensure that the cytoplasmic fraction was pure from nuclear contamination, all extractions were analyzed using capillary electrophoresis (Additional file 1: Figure S1). The nuclear and cytoplasmic preparations had, in addition to the ribosomal peaks, discriminatory signature profiles in which the nuclear fractions contained an additional peak, which were present only in the total RNA preparation [3,13]. The strongest signal, at roughly 4000 nucleotides (~52 s), was used to determine nuclear RNA presence. All of the total RNA samples displayed the signature peak, whereas the cytoplasmic fractions did not (except for one cytoplasmic U-2 OS sample, which was removed from further processing). The samples were then sequenced using massively parallel sequencing, and RPKM (Reads Per Kilobase of exon model per Million mapped reads) values were calculated [14]. Expression values were in excellent agreement between total and cytoplasmic preparations for every cell line (Pearson correlation coefficient > 0.93 (Figure 1A and Additional file 2: Figure S2) and the distribution of the RPKM values between the total and cytoplasmic fractions did not differ significantly (Figure 1B). In all cell lines, the gene coverage was slightly higher in cytoplasmic RNA than in total RNA.

**Figure 2:**
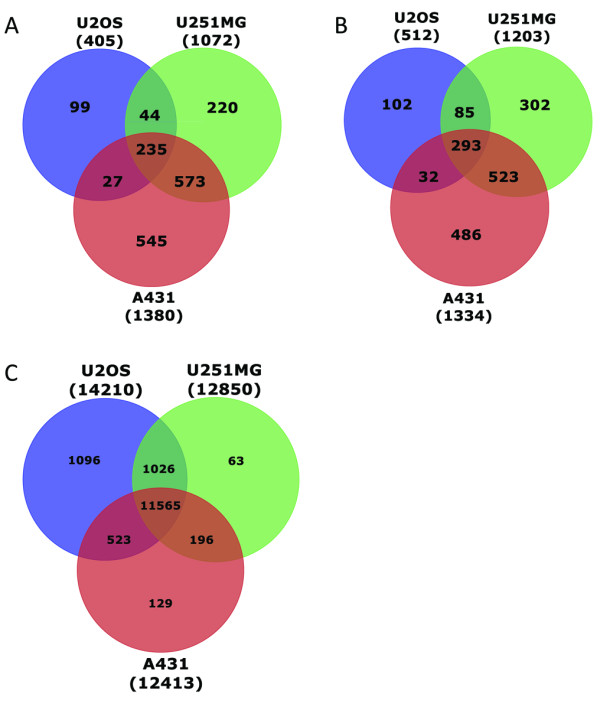
**Number of differentially detected genes between the preparation methods for each cell line. A**: Genes detected at a significantly higher level (**A**), lower level (**B**) or with no difference (**C**) in total RNA compare to cytoplasmic RNA. The percentages of differentially detected genes were: A-431 18%, U-251MG 15%, and U-2 OS 6%; calculated as the sum of genes at a higher and lower level divided by the total number of detected genes.

The DESeq algorithm was used to find sets of genes detected at different levels in cytoplasmic and in total RNA [15], hereafter referred to as *differentially detected* (DD) genes. A number of DD genes were identified between the total and cytoplasmic fractions within each cell line (Figure 2A–C). In A-431 and U-251MG, 18% and 15% of the genes were detected in different amounts between total and cytoplasmic RNA of the approximately 15000 detected genes; whereas in U-2 OS, only 6% of the genes were differentially detected (*p* < 0.001, based on three replicates for each RNA fraction). There were approximately as many genes detected at higher levels in total RNA (1380, 405, and 1072 in A-431, U-2 OS, and U-251MG, respectively) and in cytoplasmic RNA (1334, 512, and 1203 genes in A-431, U-2 OS, and U-251MG, respectively).

### Length and structure of untranslated regions influence nucleus-to-cytoplasm transportation rate of transcripts

Messenger RNAs vary in sequence and length and this can affect their rate of transportation to the cytoplasm. To investigate this, genes that were detected differentially—in one, two, or all three cell lines—were selected and classified into two groups: genes that had a higher number of copies in the total RNA fraction and genes that had a lower number of copies in the total RNA fraction and plotted separately (Figure 2A and B). Differential detection of genes in total or cytoplasmic RNA fractions relies on that total RNA fraction would contain all mature polyadenylated transcripts whether they were in the cytoplasm or in the nucleus of the cell, whereas the cytoplasmic fractions only contain transcripts already transported to the cytoplasm.

**Figure 3:**
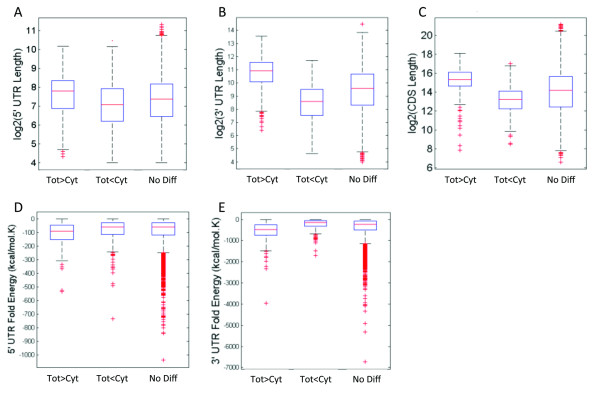
**Boxplot showing length and fold energies of UTRs and coding sequence for all cell lines.** Genes detected at a significantly higher level in total RNA than in cytoplasmic RNA (Tot>Cyt), lower level (Tot<Cyt), and genes with no significant differential detection (No Diff). **A**: Length of 5’ UTRs. **B**: Length of 3’ UTRs. **C**: Coding sequence length. **D**: Fold energy for 5’ UTRs. **E**: Fold energy for 3’ UTRs.

To study whether the lengths of untranslated regions (UTRs) could affect the transportation rate of transcripts, we compared the UTR and coding sequence lengths of differentially detected genes with those of genes exhibiting no differential detection. We found that genes detected in higher amounts in total RNA (*p* < 0.001) tended to have longer UTRs (Figure 3A and B) and that a significant proportion of longer transcripts were detected at lower levels in the cytoplasmic RNA (Figure 3C and Additional file 3 Figure S3C). This trend was consistent for genes that were differentially detected in one or more cell line (Figure 3 and Additional file 3: Figure S3A and B). We obtained UTR fold energies of 15,127 genes from the UCSC genome browser (calculated by Vienna RNA Package [16]). A more negative fold energy corresponds to a more structured sequence. Figure 3D–E and Additional file 4: Figure S4 show that genes that were detected at higher levels in total RNA had lower UTR fold energies (were more structured) than those with no differential detection. The data also show that the secondary structure of the 3’ UTRs had a stronger effect compared to 5’ UTRs.

### Genes with higher numbers of miRNA target sites were detected in lower levels in the cytoplasmic RNA fraction compare to total RNA fraction

**Figure 4:**
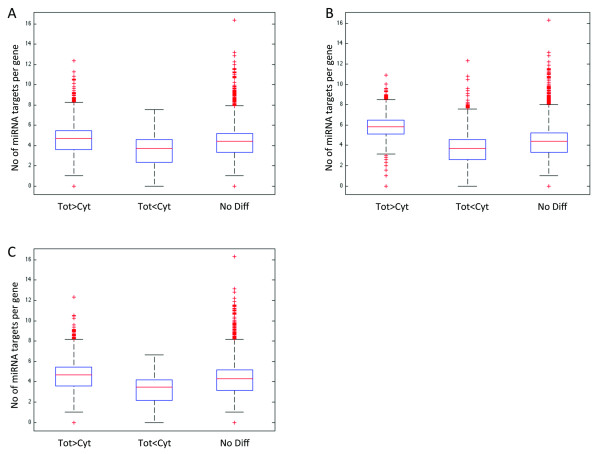
**Boxplot showing the number of microRNA targets per gene for all three cell lines separately.** Genes detected at a significantly higher level in total RNA than in cytoplasmic RNA (Tot>Cyt), lower level (Tot<Cyt), and genes with no significant differential detection (No Diff). **A**: A-431. **B**: U-2 OS. **C**: U-251 MG.

Transcripts that are degraded in the cytoplasm in high rates will also contribute to the differential detection since those degraded in cytoplasm will be detected at lower levels in the cytoplasmic RNA fraction compared to total RNA fraction. To investigate whether these genes have a higher number of micro-RNA (miRNA) targets, hence resulting in a higher probability for degradation when exported into the cytoplasm, an analysis comparing the number of miRNA targets per gene was performed. The same method to classify differentially detected genes (described in Figure 2) was used for the analysis. The miRNA data for the three cell lines (A-431, U-2 OS, U-251 MG) has been described elsewhere [17]. The list of experimentally verified miRNAs per target gene was downloaded from miRTarBase [18] and compared with the list of expressed miRNA in each cell line. As described by Akan et al., the three cell lines have very similar miRNA profiles but U-2 OS have more uniquely expressed miRNAs. There was a significant difference (p < 0,001) between the three groups of differentially detected genes for each cell line (Figure 4). The genes that had a higher number of transcripts in the total RNA fraction (Tot>Cyt) showed for all three cell lines a slightly higher number of miRNA targets than the genes that had a lower number of copies in the total RNA fraction (Tot<Cyt) and genes that where not differentially detected (No Diff). Interestingly, the cell line with a more pronounces difference between the groups, U-2 OS, is also the cell line that has more uniquely expressed miRNAs, and this could be the explanation for the slightly higher number of miRNA targets per gene seen in U-2 OS. Overall, the data suggests that the miRNA may be one of the contributing factors for differential detection of genes.

### Correlation between mRNA and protein expression

A ratio-based correlation analysis (Spearman) was performed between protein abundance levels (detected by mass spectrometry for approximately 4700 proteins)[10] and the corresponding total and cytoplasmic mRNA levels, for each cell line. For U-2 OS, the correlation coefficient between protein abundance and total RNA was 0.6717 and between protein abundance and cytoplasmic RNA was 0.6790. The correlation coefficients for the other two cell lines (U-251MG and A-431) were very similar. There were no significant differences (*p* = 0.6) between total and cytoplasmic RNA levels in terms of correlation with protein abundance. The correlations were similar whether differentially detected genes were included or excluded, see Additional file 5: Table S1 for correlation coefficients between protein abundance and total and cytoplasmic RNA, respectively, for genes detected differentially in all three cell lines.

## Discussion

When designing a gene expression experiment with the goal of measuring steady-state levels of mRNA, care should be taken to isolate RNA from the correct cellular compartment. Currently, the majority of RNA-Seq experiments sequence mature transcripts (via poly-A tail enrichment) in the total RNA fraction, which also contain mature mRNA species to some degree [19]. Removing the ~5–10 times more complex nuclear RNA [19] could reduce the overall complexity and enable deeper sampling of the remaining mRNA population and thus increase sensitivity. However, isolating the cytoplasmic RNA instead of total RNA is feasible when working with cell cultures, but for many other biological models are total RNA the only choice.

Despite the proposed advantage of sequencing only cytoplasmic RNA for cells in suspension, it is still not clear whether the cytoplasmic fraction represents the full complexity of the steady-state RNA of whole cells. One argument against using cytoplasmic RNA could be that the translation levels of certain transcripts might be regulated by their transportation rate from nucleus to cytoplasm [6,7]. Moreover, the transportation rate of transcripts from nucleus to cytoplasm could depend on particular properties of the transcript such as length or sequence.

Here, we investigated how the representations of transcripts differ between the cytoplasmic and total RNA fractions. There were 405, 1072, and 1380 transcripts in U-2 OS, U-251MG, and A-431 that were detected at higher levels in total RNA than in cytoplasmic RNA. This indicates that a significant proportion of the mature transcripts were retained in the nucleus, which then contributed to higher detection levels in the total RNA fraction since the cytoplasmic RNA lacked the mature transcripts from the nucleus. UTR fold energies can influence post-transcriptional regulation and it has been shown that UTR fold energies of mRNA transcripts are lower than those of random sequences of the same length with the same mononucleotide frequency [20,21]. Interestingly, most of the genes detected at higher level in total RNA had long and structured 5’ and 3’ UTR sequences as well as longer coding sequences, in all cell lines. Furthermore, it may cause an improper estimation of the RNA levels of these transcripts in the cytoplasmic fraction. Similarly, shorter genes or genes with shorter UTRs were overestimated in the cytoplasmic fraction. This mis-estimation could introduce biases and should be considered in the analysis of transcriptome.

Hence, our data indicates that the transportation rate of transcripts from nucleus to cytoplasm depends on the sequence features of transcripts. Selective degradation of transcripts by for example the exosome complex and the half-life of transcripts cannot be ruled out as contributing factors. The results from the comparison of microRNA targets per gene for all three cell lines show that there is a higher number of microRNA targets per gene for genes detected differentially higher in the total RNA fraction compared to the cytoplasmic RNA fraction. This could indicate that these genes are subject to degradation to a higher degree when entering the cytoplasm. However this does not explain the higher number of genes with structured 5’ UTR sequences as well as longer coding sequences in the total RNA fraction. Therefore, we propose that both nuclear retention and cytoplasmic RNA degradation via miRNAs are the main contributors to the differential detection of genes.

There were 512, 1203, and 1334 genes for U-2 OS, U-251MG, and A-431, respectively, that were detected at higher levels in cytoplasmic RNA than in total RNA. There is no obvious biological reason for this. However, a technical explanation can be suggested: owing to the lower representation of longer transcripts in the cytoplasmic fraction, there was relatively more sequencing space. This could have allowed for better coverage of shorter transcripts in cytoplasmic RNA than in total RNA. Indeed, most of the genes detected at higher levels in the cytoplasmic fraction had shorter coding lengths. However, not all the differentially detected genes were the same for all cell lines. This supports the fact that there are also cell-specific factors that affect the nuclear retention of transcripts, apart from transcript sequence and structure [7].

Our results have shown that the total and cytoplasmic fractions yield different representations of steady-state RNA levels. It can be argued that cytoplasmic polyadenylated RNA might correlate better with protein abundance levels if one assumes that the contribution of polyadenylated nuclear RNA to the steady-state mRNA levels in cytoplasm were not negligible. However, a previous study of mouse fibroblasts investigated mRNA and protein levels in relation to half-lives, transcription rates, and translational control and found that mRNA only explained around 40% of the variability in protein levels [22]. Our data show that cytoplasmic and total RNA correlated very similarly to protein abundance levels in all cell lines, and the correlation level is similar to what have previously been published [10]. This indicates that the neither nucleus-to-cytoplasm transportation rate nor the miRNA mediated degradation of transcripts affect protein abundance at a global level. However, future studies with synchronized cells and different time points would shed some more light upon the correlation between the RNA and protein population in a cell. Furthermore, including all transcripts and not only polyadenylated RNA would give amore complete overview of the RNA population in a given cell type.

## Conclusions

Overall, our findings show that there are significant differences between total mRNA and cytoplasmic mRNA, which should be considered when comparing gene and protein expression patterns, and in general when using mRNA levels in different cellular compartments as a proxy for protein levels. Such efforts include whole genome/proteome comparisons, such as the human protein atlas initiative (http://www.proteinatlas.com) as well as other global efforts that correlate disease with genomic, transcriptomic, and proteomic information. Furthermore, our findings show that expression levels derived from the total RNA fraction be regarded as an appropriate estimate of the amount of mRNAs present in a given cell population, independent of the coding sequence length or UTRs.

## Methods

### Cell cultivation

The three human cell lines—the glioblastoma cell line U-251MG (Prof. Bengt Westermark, Uppsala University), the epidermoid carcinoma cell line A-431 (DSMZ, Braunschweig, Germany), and the osteosarcoma cell line U-2 OS (ATCC-LGC, Middlesex, United Kingdom)—were cultivated at 37°C in a 5% CO_2_ environment in media suggested by the providers. Each cell-line was cultivated in four biological replicates and the cells were harvested during log-phase growth (60–70% confluency).

### RNA isolation and purification

RNA was extracted immediately after cell harvest. The total RNA fraction and the cytoplasmic fraction were extracted separately. The RNeasy Mini extraction kit (Qiagen, Hilden, Germany) was used according to the manufacturer’s instructions for total RNA isolation. Cytoplasmic RNA was isolated according to the RNeasy protocol, except that the standard lysis buffer was exchanged for the lysis buffer RNL [50 mM Tris–HCl, pH 8.0; 140 nM NaCl; 1.5 mM MgCl2; 0.5% (v/v) Nonidet P-40 (1.06 g/ml); and 1 mM DTT added just before use]. The extracted RNA samples were analyzed using an Experion automated electrophoresis system (Bio-Rad Laboratories, Hercules, CA, USA) with the standard-sensitivity RNA chip. All of the total RNA samples displayed a total RNA signature peak; as did one replicate of the cytoplasmic fraction of U-2 OS, which was discarded.

### Library preparation for sequencing

Each cell-line was prepared in quadruplicate, with four biological replicates for total RNA and four for cytoplasmic RNA. A total of 3 μg of high-quality RNA (RNA integrity number = 10) per sample was used as input material for the mRNA sample preparations. The concentration and the RNA integrity number of the samples were determined from the run with the standard-sensitivity RNA chip on the Experion automated electrophoresis system (Bio-Rad Laboratories, Hercules, CA, USA). The samples were bar-coded and prepared according to the protocol (Cat# RS-930-1001) of the manufacturer (Illumina, San Diego, CA, USA) with the automated platform described previously [23].

### Clustering and sequencing

The bar-coded libraries were clustered on a cBot cluster-generation system using an Illumina HiSeq single-read cluster-generation kit according to the manufacturer’s instructions. The libraries were pooled together two and two in equal concentration for each lane on the flow cell, and sequenced as single reads to 100 bp on an Illumina HiSeq 2000. All lanes were spiked with 1% phiX control library. The sequencing run was performed according to the manufacturer’s instructions and generated a total of 908 million reads with a median of 40 million reads per sample and replicate that passed the Illumina Chastity filter; these reads were included in the study (Additional file 6: Table S2).

### Sequence analysis

All sequences were aligned to the human genome reference hg19 with tophat [24,25] version 1.1.4 and samtools [26] version 0.1.8 using tophat standard parameters except for: --solexa1.3-quals -p 8 --GTF Homo_sapiens.GRCh37.59.gtf. Annotations from ensembl and RefSeq, downloaded from UCSC Genome Browser, were used to assign features to genomic positions. Sequences aligned to the human genome were assigned to features and counted by HTSeq version 0.4.6 with parameters: -m intersection-strict -s no -t exon (Additional file 7: Table S3). The R/Bioconductor package DESeq [15] was used to call differential gene expression on counts generated by HTSeq. All biological replicates had *R*^*2*^ (Spearman) correlation of gene expression (read counts) greater than 0.94.

Reads per kilobase of exon per million mapped sequence reads (RPKM) values for features were calculated by rpkmforgenes.py using the parameters: -sam -gffann –readcount. Estimations of intergenic expression levels for each replicate were calculated by rpkmforgenes.py and the R script cut_off.1.0.R (Additional file 8: Table S4) [27].

Reads were trimmed to determine the effect of sequencing length on the number of called differentially expressed genes using a custom perl script: trim_length.pl, which is available on github (https://github.com/henrikstranneheim).

Analysis of gene categories and pathways was performed by WebGestalt2 [28] with parameters: Id Type: ensembl_gene_stable_id, Ref Set: entrezgene, Significance Level: Top10, Statistics Test: Hypergeometric, MTC: BH, Minimum: 2.

5’ and 3’ UTR lengths and coding sequences were downloaded from UCSC. Lengths and fold energies were calculated with the Vienna RNA Package [16].

### Mass spectrometry data

The protein data used in this study were generated in a previous study by Lundberg *et al*. [10], where a deep proteomic analysis was performed on the same three cell lines (A-431, U-2 OS, and U-251MG) used in this study. The cell lines were cultivated with amino acids with different isotopes and analyzed by mass spectrometry using a triple-SILAC method [29-31].

## Abbreviations

bp: base pair; CDS: Coding sequence; DD: Differentially detected; miRNA: micro-RNA; RPKM: Reads per kilobase of exon model per million mapped reads; UTRs: Untranslated regions.

## Competing interests

The authors declare that they have no competing interests.

## Author’s contributions

BWS participated in the design of the study, purified the RNA, prepared the Illumina mRNA sequencing libraries, performed bioinformatics, and participated in drafting the manuscript. HS participated in the design of the study, performed bioinformatics and statistical analyses, and drafted the manuscript. . JH did the miRNA preparation. MK contributed with reagents, materials, and analysis tools. EL contributed with the cell lines and the growth of the cells. PA performed bioinformatics and statistical analyses and participated in drafting the manuscript. JL participated in the design of the study and participated in drafting the manuscript. All authors read and approved the final manuscript.

## Supplementary Material

Additional file 1 **Figure S1. **Overlays of electrophoresis diagrams of purified RNA. Total RNA and cytoplasmic RNA, showing the two ribosomal peaks (18S and 28S). In addition, the total RNA contains a nucleus-specific peak at roughly 4000 nucleotides (~52 s), marked with an arrow in the upper diagram, which is missing in the cytoplasmic RNA fraction. Click here for file

Additional file 2 **Figure S2. **Scatter plots of gene expression levels between total and cytoplasmic RNA. The median of the gene expression values of total and cytoplasmic RNA. The Pearson correlation coefficient R^2^ is displayed in each scatter plot. A: U-2 OS, B: U-251 MG, C: A-431. Click here for file

Additional file 3 **Figure S3.** Boxplot showing the length and coding sequence for all three cell lines. Genes detected at a significantly higher level in total RNA than in cytoplasmic RNA (Tot>Cyt), lower level (Tot<Cyt), and genes with no significant differential detection (No Diff). A: Length of 5’ UTRs. B: Length of 3’ UTRs. C: Coding sequence length. Click here for file

Additional file 4 **Figure S4.** Boxplot showing the fold energies of UTRs for all three cell lines. Genes detected at a significantly higher level in total RNA than in cytoplasmic RNA (Tot>Cyt), lower level (Tot<Cyt), and genes with no significant differential detection (No Diff). A: Fold energy of 5’ UTRs. B: Fold energy of 3’ UTRs. Click here for file

Additional file 5 **Table S1.** Spearman correlation for differentially and not differentially detected genes in all three cell lines. Click here for file

Additional file 6 **Table S2.** Summary of information from the sequencing run. Click here for file

Additional file 7 **Table S3. **Reads assigned to features using HTSeq. Click here for file

Additional file 8 **Table S4.** Estimation of intergenic expressions levels within replicates based on RPKM. Click here for file

## References

[B1] Shendure J The beginning of the end for microarrays? Nat Methods 2008 5 7 585 587 10.1038/nmeth0708-585 18587314

[B2] Mortazavi A Williams BA McCue K Schaeffer L Wold B Mapping and quantifying mammalian transcriptomes by RNA-Seq Nat Methods 2008 5 7 621 628 10.1038/nmeth.1226 18516045 PMC13303166

[B3] Trask HW Cowper-Sal-lari R Sartor MA Gui J Heath CV Renuka J Higgins AJ Andrews P Korc M Moore JH Microarray analysis of cytoplasmic versus whole cell RNA reveals a considerable number of missed and false positive mRNAs RNA 2009 15 10 1917 1928 10.1261/rna.1677409 19703940 PMC2743046

[B4] Hieronymus H Silver PA Genome-wide analysis of RNA-protein interactions illustrates specificity of the mRNA export machinery Nat Genet 2003 33 2 155 161 10.1038/ng1080 12524544

[B5] Garcia-Martinez J Aranda A Perez-Ortin JE Genomic run-on evaluates transcription rates for all yeast genes and identifies gene regulatory mechanisms Mol Cell 2004 15 2 303 313 10.1016/j.molcel.2004.06.004 15260981

[B6] Prasanth KV Prasanth SG Xuan Z Hearn S Freier SM Bennett CF Zhang MQ Spector DL Regulating gene expression through RNA nuclear retention Cell 2005 123 2 249 263 10.1016/j.cell.2005.08.033 16239143

[B7] Yasuda Y Miyamoto Y Yamashiro T Asally M Masui A Wong C Loveland KL Yoneda Y Nuclear retention of importin alpha coordinates cell fate through changes in gene expression EMBO J 2012 31 1 83 94 21964068 10.1038/emboj.2011.360 PMC3252573

[B8] Chen CY Gherzi R Ong SE Chan EL Raijmakers R Pruijn GJ Stoecklin G Moroni C Mann M Karin M AU binding proteins recruit the exosome to degrade ARE-containing mRNAs Cell 2001 107 4 451 464 10.1016/S0092-8674(01)00578-5 11719186

[B9] Schaeffer D Tsanova B Barbas A Reis FP Dastidar EG Sanchez-Rotunno M Arraiano CM van Hoof A The exosome contains domains with specific endoribonuclease, exoribonuclease and cytoplasmic mRNA decay activities Nat Struct Mol Biol 2009 16 1 56 62 10.1038/nsmb.1528 19060898 PMC2615074

[B10] Lundberg E Fagerberg L Klevebring D Matic I Geiger T Cox J Algenas C Lundeberg J Mann M Uhlen M Defining the transcriptome and proteome in three functionally different human cell lines Mol Syst Biol 2010 6 450 21179022 10.1038/msb.2010.106 PMC3018165

[B11] de Sousa Abreu R Penalva LO Marcotte EM Vogel C Global signatures of protein and mRNA expression levels Mol Biosyst 2009 5 12 1512 1526 20023718 10.1039/b908315d PMC4089977

[B12] Maier T Guell M Serrano L Correlation of mRNA and protein in complex biological samples FEBS Lett 2009 583 24 3966 3973 10.1016/j.febslet.2009.10.036 19850042

[B13] Wang Y Zhu W Levy DE Nuclear and cytoplasmic mRNA quantification by SYBR green based real-time RT-PCR Methods 2006 39 4 356 362 10.1016/j.ymeth.2006.06.010 16893657

[B14] Ramskold D Wang ET Burge CB Sandberg R An abundance of ubiquitously expressed genes revealed by tissue transcriptome sequence data PLoS Comp Biol 2009 5 12 e1000598 10.1371/journal.pcbi.1000598 PMC2781110 20011106

[B15] Anders S Huber W Differential expression analysis for sequence count data Genome Biol 2010 11 10 R106 10.1186/gb-2010-11-10-r106 20979621 PMC3218662

[B16] Hofacker IL Vienna RNA secondary structure server Nucleic Acids Res 2003 31 13 3429 3431 10.1093/nar/gkg599 12824340 PMC169005

[B17] Akan P Costea PI Alexeyenko A Hedberg L Werne Solnestam B Lundin S Hällman J Lundberg E Uhlén M Lundeberg J A Comprehensive Analysis of the Genome, Transcriptome and Proteome Landscapes of Three Human Tumor Cell Lines Reveals That Genomic Alterations Function Co-operatively in Tumorigenesis 2012 Submitted 10.1186/gm387 PMC3580420 23158748

[B18] Hsu SD Lin FM Wu WY Liang C Huang WC Chan WL Tsai WT Chen GZ Lee CJ Chiu CM miRTarBase: a database curates experimentally validated microRNA-target interactions Nucleic Acids Res 2011 39 Database issue D163 169 21071411 10.1093/nar/gkq1107 PMC3013699

[B19] Sippel AE Hynes N Groner B Schutz G Frequency distribution of messenger sequences within polysomal mRNA and nuclear RNA from rat liver Eur J Biochem 1977 77 1 141 151 10.1111/j.1432-1033.1977.tb11652.x 71236

[B20] Clote P Ferre F Kranakis E Krizanc D Structural RNA has lower folding energy than random RNA of the same dinucleotide frequency RNA 2005 11 5 578 591 10.1261/rna.7220505 15840812 PMC1370746

[B21] Ringner M Krogh M Folding free energies of 5'-UTRs impact post-transcriptional regulation on a genomic scale in yeast PLoS Comp Biol 2005 1 7 e72 10.1371/journal.pcbi.0010072 PMC1309706 16355254

[B22] Schwanhausser B Busse D Li N Dittmar G Schuchhardt J Wolf J Chen W Selbach M Global quantification of mammalian gene expression control Nature 2011 473 7347 337 342 10.1038/nature10098 21593866

[B23] Stranneheim H Werne B Sherwood E Lundeberg J Scalable transcriptome preparation for massive parallel sequencing PLoS One 2011 6 7 e21910 10.1371/journal.pone.0021910 21760920 PMC3131396

[B24] Langmead B Trapnell C Pop M Salzberg SL Ultrafast and memory-efficient alignment of short DNA sequences to the human genome Genome Biol 2009 10 3 R25 10.1186/gb-2009-10-3-r25 19261174 PMC2690996

[B25] Trapnell C Pachter L Salzberg SL TopHat: discovering splice junctions with RNA-Seq Bioinformatics 2009 25 9 1105 1111 10.1093/bioinformatics/btp120 19289445 PMC2672628

[B26] Li H Handsaker B Wysoker A Fennell T Ruan J Homer N Marth G Abecasis G Durbin R The Sequence Alignment/Map format and SAMtools Bioinformatics 2009 25 16 2078 2079 10.1093/bioinformatics/btp352 19505943 PMC2723002

[B27] Ramskold D Wang ET Burge CB Sandberg R An abundance of ubiquitously expressed genes revealed by tissue transcriptome sequence data PLoS Comput Biol 2009 5 12 e1000598 10.1371/journal.pcbi.1000598 20011106 PMC2781110

[B28] Zhang B Kirov S Snoddy J WebGestalt: an integrated system for exploring gene sets in various biological contexts Nucleic Acids Res 2005 33 Web Server issue W741 748 15980575 10.1093/nar/gki475 PMC1160236

[B29] Wisniewski JR Zougman A Mann M Combination of FASP and StageTip-based fractionation allows in-depth analysis of the hippocampal membrane proteome J Proteome Res 2009 8 12 5674 5678 10.1021/pr900748n 19848406

[B30] Wisniewski JR Zougman A Nagaraj N Mann M Universal sample preparation method for proteome analysis Nat Methods 2009 6 5 359 362 10.1038/nmeth.1322 19377485

[B31] Shevchenko A Tomas H Havlis J Olsen JV Mann M In-gel digestion for mass spectrometric characterization of proteins and proteomes Nat Protoc 2006 1 6 2856 2860 17406544 10.1038/nprot.2006.468

