# Supplementary figures and images for: Comparison of total and cytoplasmic mRNA reveals global regulation by nuclear retention and miRNAs
Source: BMC Genomics. 2012 Oct 30;13:574. doi: 10.1186/1471-2164-13-574 (PMC3495644; doi:10.1186/1471-2164-13-574)

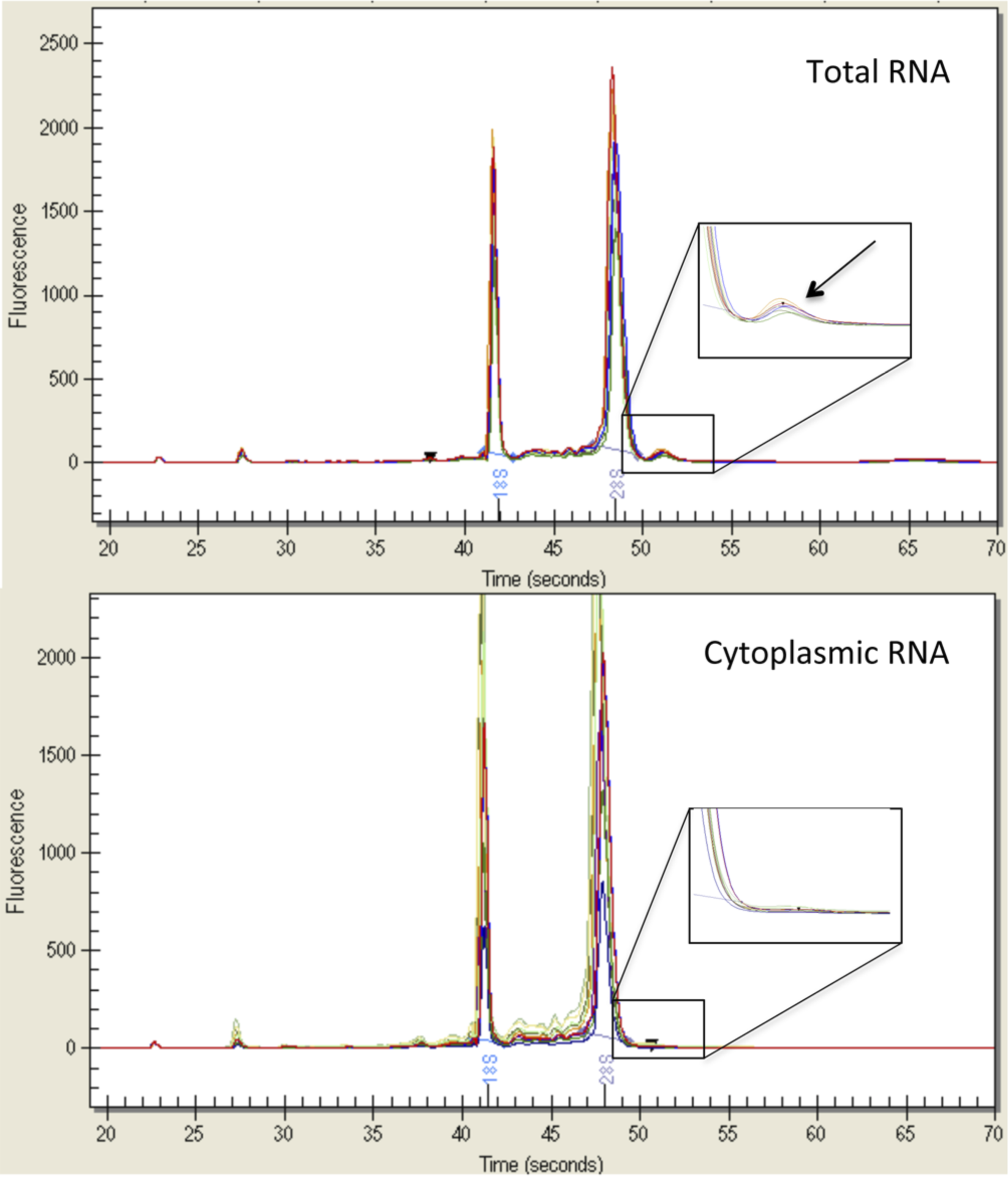

Supplement: Additional file 1 — Figure S1. Overlays of electrophoresis diagrams of purified RNA. Total RNA and cytoplasmic RNA, showing the two ribosomal peaks (18S and 28S). In addition, the total RNA contains a nucleus-specific peak at roughly 4000 nucleotides (~52 s), marked with an arrow in the upper diagram, which is missing in the cytoplasmic RNA fraction. [file 1471-2164-13-574-S1.tiff]

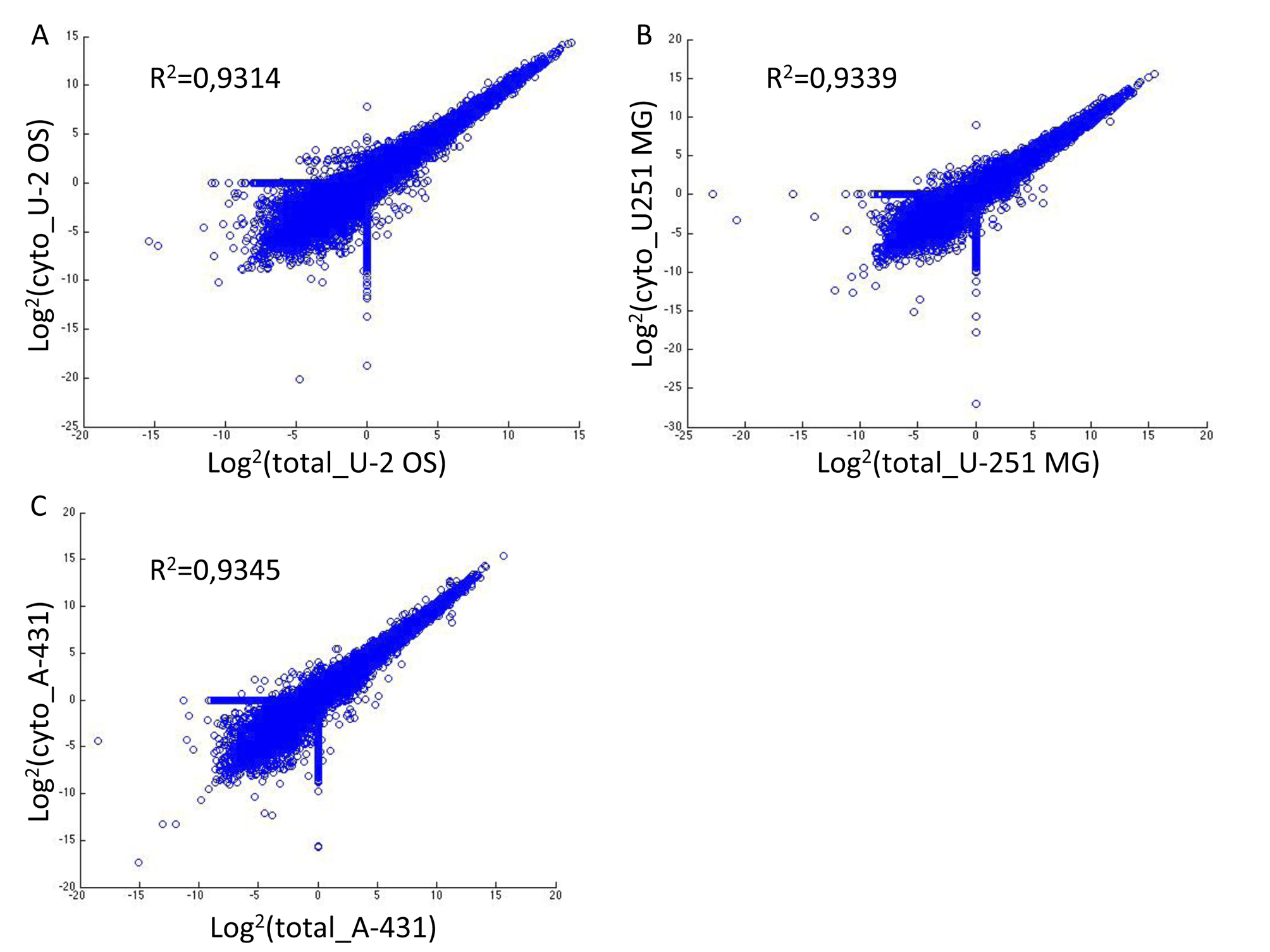

Supplement: Additional file 2 — Figure S2. Scatter plots of gene expression levels between total and cytoplasmic RNA. The median of the gene expression values of total and cytoplasmic RNA. The Pearson correlation coefficient R2 is displayed in each scatter plot. A: U-2 OS, B: U-251 MG, C: A-431. [file 1471-2164-13-574-S2.tiff]

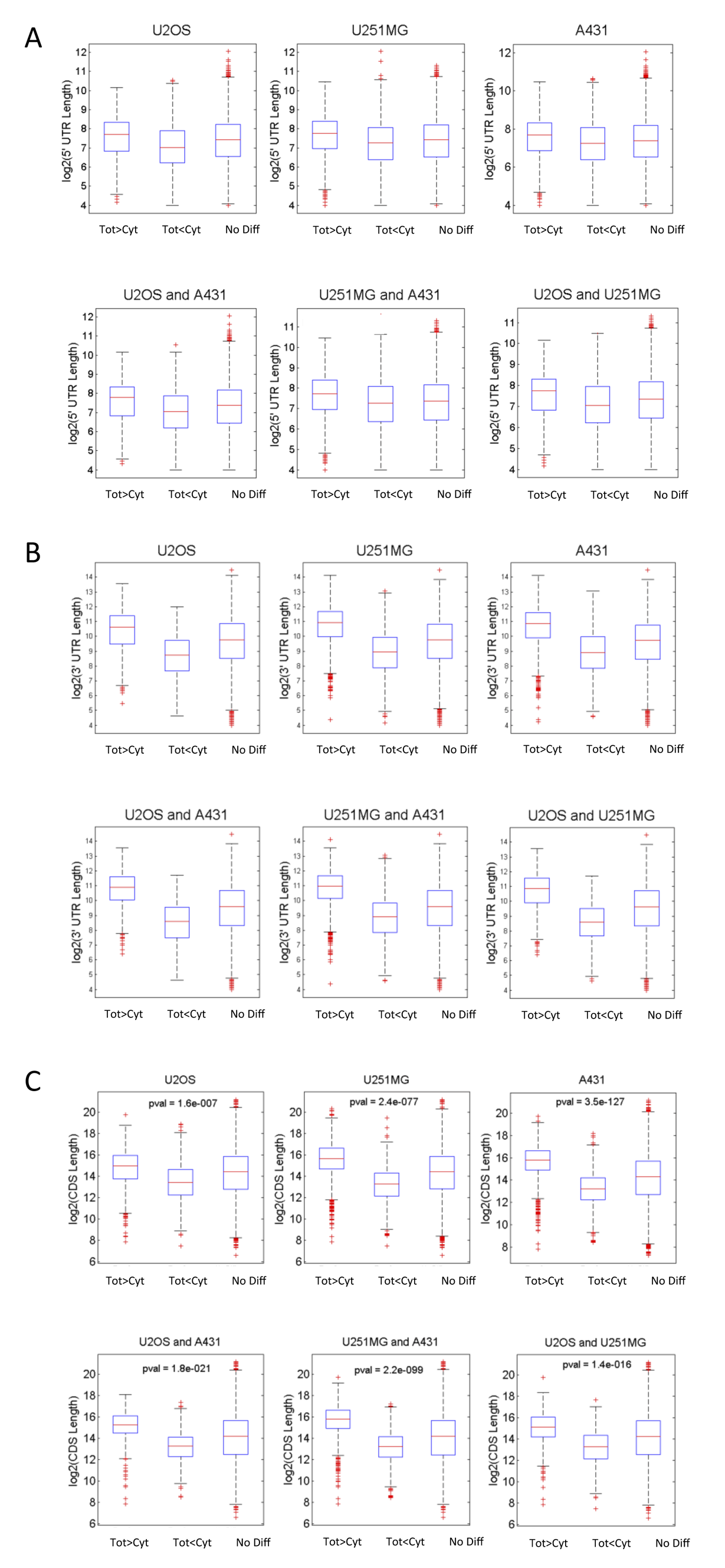

Supplement: Additional file 3 — Figure S3. Boxplot showing the length and coding sequence for all three cell lines. Genes detected at a significantly higher level in total RNA than in cytoplasmic RNA (Tot>Cyt), lower level (Tot<Cyt), and genes with no significant differential detection (No Diff). A: Length of 5’ UTRs. B: Length of 3’ UTRs. C: Coding sequence length. [file 1471-2164-13-574-S3.tiff]

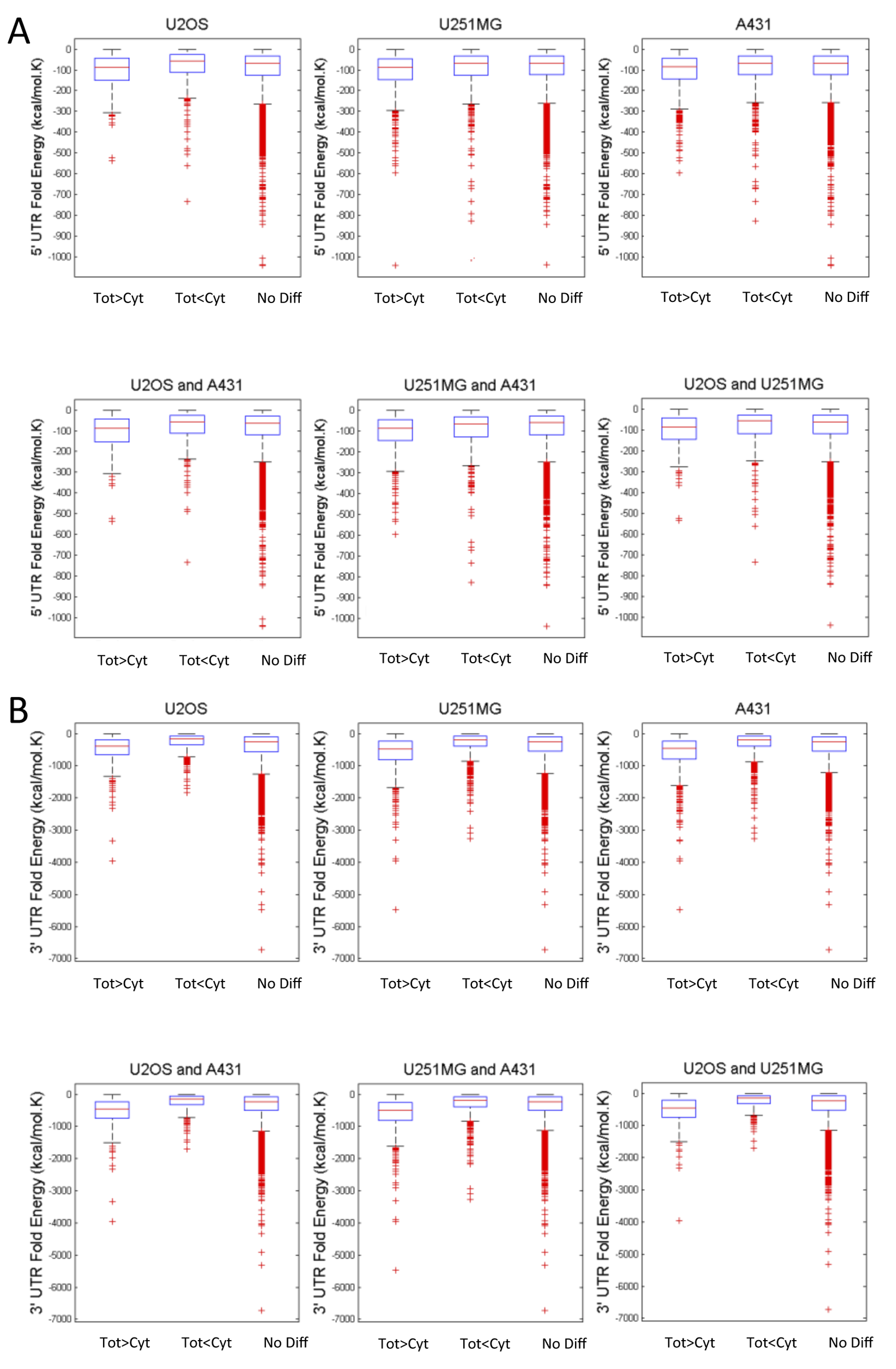

Supplement: Additional file 4 — Figure S4. Boxplot showing the fold energies of UTRs for all three cell lines. Genes detected at a significantly higher level in total RNA than in cytoplasmic RNA (Tot>Cyt), lower level (Tot<Cyt), and genes with no significant differential detection (No Diff). A: Fold energy of 5’ UTRs. B: Fold energy of 3’ UTRs. [file 1471-2164-13-574-S4.tiff]
